# Complications Associated with Microdebriders in Otolaryngology Procedures from 2011 to 2021: A MAUDE Study

**DOI:** 10.1002/oto2.83

**Published:** 2023-10-19

**Authors:** Hari Magge, Esther Lee, Timothy B. Shaver, Punam G. Thakkar, Ameet Singh

**Affiliations:** ^1^ Division of Otolaryngology‐Head and Neck Surgery George Washington University School of Medicine & Health Sciences Washington District of Columbia USA

**Keywords:** adverse events, endoscopic sinus surgery, MAUDE, microdebrider, patient safety

## Abstract

**Objective:**

The microdebrider is a powered surgical instrument that is widely used in the field of otolaryngology. We aim to identify the type and frequency of device malfunctions, patient complications, and subsequent interventions related to the use of microdebriders.

**Study Design:**

Cross‐sectional analysis.

**Setting:**

The US Food and Drug Administration (FDA) 2011 to 2021 Manufacturer and User Facility Device Experience (MAUDE) database.

**Methods:**

The MAUDE database was queried for reports of “microdebrider,” with adverse events selected that pertained to usage in head and neck surgeries from January 1, 2011 to December 31, 2021.

**Results:**

There were 282 adverse events in 267 individual medical device reports (MDR). Although the majority of the reports did not specify the specific operation, endoscopic sinus surgery was the most common reported procedure (89, 33.3%). The most common cause of device malfunction was due to a broken piece (120, 48.2%) followed by overheating of the microdebrider motor (78, 31.3%). Of the reports which specified patient injury, the most commonly reported was “unintentional tissue damage,” (10, 32.3%).

**Conclusion:**

The microdebrider has demonstrated utility within the field of otolaryngology, but is not without risk of malfunction that can cause patient injury. By understanding possible risks of microdebrider usage, including tissue damage, burns, and bleeds caused by device malfunction or operator error, physicians can be better prepared to address complications and educate patients.

The microdebrider is a powered instrument with significant importance in an otolaryngologist's arsenal. Its initial conception was that of a “rotary vacuum shaver” in the late 1960s for the resection of acoustic neuromas.^1^ Subsequently used by orthopedists for arthroscopic surgery, microdebriders made their way into the otolaryngology realm in the mid‐1990s, and have remained since that time.^2^ Mainly used in endoscopic sinus surgery, the microdebrider's otolaryngologic implications extend to tonsillectomy and adenoidectomy along with laryngeal surgery in order to remove nodules or lesions.^3^, ^4^, ^5^, ^6^ The microdebrider consists of three primary parts: the console which provides the power source, a handpiece, and a cutting instrument, including blades and burs (Figure 1).^7^ The cutting instrument attaches to the handpiece and consists of two hollow cylinders, one inside the other. At the distal end of the device, the outer cylinder has a port allowing for tissue to enter, where the inner cylinder, with its cutting apparatus, shears the tissue. Suction is employed in these surgical cases to allow for effective removal of tissue through the device and into a “trap” via tubing attached to the handpiece, allowing the surgical field to be unobstructed from blood and resected tissue. Surgeons have a variety of disposable and reusable blades that can adjust the frequency at which the microdebrider operates. The two primary motions include rotation and oscillation, each ideal for specific tissue removal.

**Figure 1:**
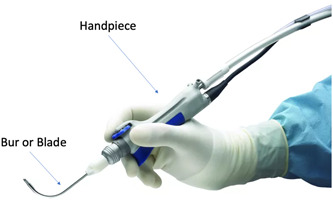
Image of microdebrider.

The microdebrider is very useful in procedures involving the nasal cavity, oropharynx, and larynx. However, since the microdebrider is a powered instrument that is frequently used in such confined spaces, usage can result in patient harm with user error or device malfunction. Several studies have identified complications of microdebrider use within the head and neck, but conclusions have largely been derived from single institutional data and focused on a single head and neck subsite.^8^, ^9^, ^10^

Our study utilized the US Food and Drug Administration's (FDA) Manufacturer and User Facility Device Experience (MAUDE) Database to report the incidence and type of complications that occur with microdebrider usage in otolaryngologic surgeries. The MAUDE Database is an online resource that compiles the adverse events of medical devices. Medical device reports (MDRs) include suspected device‐associated malfunctions, injuries, and deaths. The database includes those who are required to report (manufacturers, importers, and device user facilities) as well as voluntary reporters (physicians, other health care professionals, patients, and consumers). Thus, the MAUDE database is a passive surveillance system that is limited by potentially incomplete, inaccurate, or biased data. Due to underreporting, data retrieved from the database cannot be used to determine the incidence or prevalence of an event. Nevertheless, the MAUDE database represents a valuable resource for surveillance of medical devices and provides substantial data that may supplement existing knowledge. To date, there appears to be no study that has utilized the MAUDE database to analyze and quantify the type and frequency of adverse events for microdebriders in otolaryngologic surgery.

## Methods

The MAUDE database was searched from January 1, 2011 to December 31, 2021 using the term “microdebrider.” Exclusion criteria included any duplicate adverse reports, cases based on the publications in the literature, and those in which microdebriders were used outside of an otolaryngological procedure (ie, orthopedic procedures). Individual reports that were submitted to the MAUDE database were defined as an MDR. An adverse event was defined as either a device malfunction, patient/operator injury, or death. Variables recorded for data analysis included the specific surgical procedure, type of microdebrider malfunction, whether the malfunction resulted in patient injury, whether the malfunction resulted in operator injury, specific location of patient injury, and any necessary intervention following the adverse event.

Device malfunction was defined as any event in which the microdebrider did not function as it was intended. The specific types of malfunctions included broken pieces of the microdebrider, navigation system malfunction, motor overheating, power malfunction, inability to clean residuals, and inadvertent scratch on the endoscopic camera caused by contact between a microdebrider and camera lens. Patient injuries that were due to the device malfunction included burn injury, orbital injury, cerebrospinal fluid leaks, arterial injury, pneumothorax, and unintentional tissue damage. Location of burn injuries included soft palate, lip, nasal floor, nostril, abdomen, and groin. In some cases, both an adverse event to the patient and a device malfunction were extracted from the same MDR. Thus, the total number of adverse events is greater than the number of reports extracted from the MAUDE database.

Root causes of device malfunctions and patient injury were categorized as defective device, operator error, or both if it was explicitly stated. The intervention required during the procedure was also recorded, if specified in the event description.

This study does not require Institutional Review Board review because it is based on publicly available deidentified data.

## Results

A total of 348 MDRs were captured in a search for “microdebrider” on the MAUDE database. Implementing exclusion criteria, 30 were removed due to being duplicates, 18 were removed due to being research articles, and 33 were removed because they were not related to an otolaryngological case. As a result, 267 MDRs with a total of 281 adverse events from December 27, 2011 through October 7, 2021 were used for data analysis. The majority of MDRs did not report the specific procedure that was being performed (N = 158, 59.2%). Functional Endoscopic Sinus Surgery (FESS) was the most commonly reported procedure (N = 89, 33.3%) followed by laryngotracheal mass excision (N = 10, 3.7%). The full list of surgical procedures is provided in Table 1.

**Table 1:** Medical Device Reports (MDRs) by Surgery Type (N = 267)

	N	%
Unspecified	158	59.2
Functional endoscopic sinus surgery	89	33.3
Laryngotracheal mass excision	10	3.7
Dacryocystorhinostomy	2	0.7
Transsphenoidal surgery	2	0.7
Otologic procedures	2	0.7
Mass excision of the cavum	1	0.4
Maxillectomy	1	0.4
Tonsillectomy and adenoidectomy	1	0.4
Turbinoplasty	1	0.4
Total	267	100.0

Approximately 89% of the adverse events were related to device malfunction. The most common device malfunction was a broken piece on the microdebrider (120, 48.2%) followed by overheating of the microdebrider motor (78, 31.3%). Of the broken pieces, the bur was the most commonly broken component (73, 29.3%), followed by the blade (45, 18.1%) and the hand piece (2, 0.8%). Of the 31 MDR related to patient injury, the most commonly reported injury was unintentional tissue damage (10, 32.3%), followed by arterial injury and burn injury (6, 19.4% each). The only operator injury reported was mild electric shock while using the handpiece. Adverse events were categorized in Table 2.

**Table 2:** Adverse Events by Category (N = 281)

	N	%
*Adverse events*		
Broken pieces	120	48.2
Bur	73	29.3
Blade	45	18.1
Handpiece	2	0.8
Navigation system malfunction	37	14.9
Motor overheating	78	31.3
Power malfunction	11	4.4
Inability to clean residuals	1	0.4
Scratched endoscopic camera	2	0.8
Total	249	88.6
*Patient injury*		
Burn injury	6	19.4
Orbital injury	4	12.9
Cerebrospinal fluid leak	4	12.9
Arterial injury	6	19.4
Pneumothorax	1	3.2
Unintentional tissue damage	10	32.3
Total	31	11.0
*Operator injury*		
Electric shock	1	100.0
Total	1	0.4
Total	281	100.0

The cause and sequelae of the device malfunction was extracted only if explicitly specified, and these results are shown in Table 3. Most of the malfunctions were due to a defective device (186, 74.7%), followed by operator error (30, 12.0%). Twenty‐one cases (8.4%) were related to both defective device and operator error. As a result of malfunction, the microdebrider required repair in 81 (32.5%) of the adverse events. There were 13 (5.2%) patient injuries reported as the result of device malfunction with 4 patients requiring further medical treatment.

**Table 3:** Cause and Sequelae of Device Malfunction

		Cause of event						Resultant injury		Intervention							
	Total	Defective device		Operator error		Both		Patient injury		Revision procedure		System analysis		Device repair		Medical treatment	
Category	N	N	%	N	%	N	%	N	%	N	%	N	%	N	%	N	%
Broken pieces																	
Bur	74	40	54.1	16	21.6	11	14.9	5	6.8	1	1.4	0	0.0	14	18.9	3	4.1
Blade	44	28	63.6	11	25.0	5	11.4	1	2.3	1	2.3	0	0.0	2	4.5	0	0.0
Handpiece	2	0	0.0	0	0.0	0	0.0	0	0.0	0	0.0	0	0.0	0	0.0	0	0.0
Navigation system malfunction	37	32	86.5	1	2.7	3	8.1	2	5.4	2	5.4	7	18.9	9	24.3	0	0.0
Motor overheating	78	76	97.4	0	0.0	1	1.3	4	5.1	1	1.3	0	0.0	53	67.9	1	1.3
Electrical system malfunction	11	10	90.9	0	0.0	1	9.1	1	9.1	1	9.1	0	0.0	3	27.3	0	0.0
Inability to clean residuals	1	0	0.0	0	0.0	0	0.0	0	0.0	0	0.0	0	0.0	0	0.0	0	0.0
Damaged camera from cleaning	2	0	0.0	2	100.0	0	0.0	0	0.0	0	0.0	0	0.0	0	0.0	0	0.0
Total	249	186	74.7	30	12.0	21	8.4	13	5.2	6	2.4	7	2.8	81	32.5	4	1.6

The cause of patient injury was due to operator error in 8 cases (25.8%) followed by both operator error and defective device in 4 cases (12.9%). Approximately 20% (6) of patients required medical treatment. The one report of operator injury from electric shock was due to a defective device (Table 4).

**Table 4:** Causes and Sequelae of Patient and Operator Injury

		Cause of event						Intervention					
	Total	Defective device		Operator error		Both		Revision procedure		Medical treatment		Device repair	
Category	N	N	%	N	%	N	%	N	%	N	%	N	%
*Patient injury*													
Burn injury	6	0	0.0	3	50.0	1	16.7	1	16.7	4	66.7	0	0.0
Orbital injury	4	1	25.0	1	25.0	1	25.0	3	75.0	0	0.0	1	25.0
Cerebrospinal fluid leak	4	0	0.0	0	0.0	1	25.0	0	0.0	1	25.0	0	0.0
Bleeding	6	0	0.0	1	16.7	0	0.0	3	50.0	0	0.0	1	16.7
Pneumothorax	1	0	0.0	0	0.0	0	0.0	0	0.0	1	100.0	0	0.0
Unintentional tissue damage	10	0	0.0	3	30.0	1	10.0	4	40.0	0	0.0	0	0.0
Total	31	1	3.2	8	25.8	4	12.9	11	35.5	6	19.4	2	6.5
*Operator injury*													
Electric shock	1	1	100.0	0	0.0	0	0.0	0	0.0	0	0.0	1	100.0
Total	1	1	100.0	0	0.0	0	0.0	0	0.0	0	0.0	1	100.0

Reported patient injuries were further specified by location of injury. The most injured site was larynx/trachea (10, 32.2%) followed by the nose, eye, and dura/skull (Table 5).

**Table 5:** Location of Patient Injury (N = 31)

	N	%
Larynx, trachea	10	32.2
Nose	4	12.9
Eye	4	12.9
Dura/skull	4	12.9
Lip	2	6.5
Hard/soft palate	1	3.2
Lingual artery	1	3.2
Brachiocephalic artery	1	3.2
Groin	1	3.2
Lung	1	3.2
Abdomen	1	3.2
Unspecified	1	3.2
Total	31	100.0

## Discussion

Microdebriders are used extensively in a variety of otolaryngological procedures, including FESS, laryngotracheal procedures, tonsillectomies, and more.^3^, ^4^, ^5^, ^6^ Microdebriders offer several advantages that make them attractive devices. One study found that microdebriders result in operating times that are 37% shorter in FESS procedures and scored significantly higher in most aspects of user friendliness.^11^ Microdebrider‐assisted inferior turbinate reduction surgeries have been shown to have better long‐term nasal obstruction and nasal airflow outcomes when compared to patients who undergo radiofrequency‐assisted procedures.^12^ Intracapsular tonsillectomies using microdebriders have also been shown to reduce short‐term pain and hasten resumption to normal activity in patients when compared to those who underwent intracapsular tonsillectomy without microdebrider.^6^ Additionally, use of microdebriders is associated with reduced aerosol generation when compared to CO_2_ laser procedures, thus reducing risk of airborne infections such as COVID‐19.^13^, ^14^

Use of microdebriders does carry risk of adverse events. Howell et al conducted an email survey regarding use of microdebriders in laryngological surgeries and described anecdotal incidents of major vocal fold scar, airway compromise, severe hemorrhage, and unintentional tissue loss related to microdebrider usage.^10^ However, to our knowledge, this is the first study to analyze adverse events associated with microdebriders across different types of surgical procedures using the MAUDE database. We believe that this knowledge will help set expectations and demonstrate risks for providers who use microdebriders in practice.

Our study found that the vast majority of medical device reports that specified surgery type were related to FESS (81.6% of reports with specific surgery type). FESS is utilized in management of acute, chronic, and recurrent sinusitis and can help improve drainage of the sinuses, resulting in symptomatic improvement.^15^ Reported complication rates of FESS have varied, but have ranged from less than 0.5% up to 3%.^16^, ^17^, ^18^ Feared major complications of FESS with microdebrider, and of microdebrider use in general, include arterial bleeds, skull base injury, and intraorbital injury.^18^ Major ophthalmic damage is possible due to the proximity of the medial rectus and optic nerve to the paranasal sinuses and the rapidity of damage following activation of the microdebrider on the periorbita.^19^ Indeed, several patients in our analysis had serious complications that had to be corrected with medical management or further surgical intervention. These complications included tissue damage, burns, arterial bleeds, cerebrospinal fluid leak, orbital damage, and pneumothorax.

Although such injuries to patients are feared complications of microdebrider use, our analysis showed that most reported microdebrider malfunctions were not associated with patient injury. Microdebriders have components that allow them to be personalized to a provider's specific use. Such technologies include specialty handpieces and blades, increased rotational speeds, incorporation of navigation systems, adaptations for specific surgery types, disposable components tailored to in‐office use, and more.^4^ Based on our findings, these customizable components are most frequently implicated in microdebrider malfunction. For example, the most common device malfunction was a broken piece, with broken burs, blades, and handpieces constituting most of these breaks. The next most common device malfunctions were motor overheatings and navigation system malfunctions.

Overall, 74.7% of reported adverse events were primarily attributable to a defective device rather than to operator error. However, when considering only broken pieces such as burs and blades, this rate drops to 54.1% and 63.6%, respectively. The remainder of cases were either fully or partially caused by operator error. For example, excess pressure placed on a blade or bur increases risk of breakage and bleeding in patients. Thus, provider education and training on how to properly use microdebriders may help avoid patient injuries.

Our findings may be useful in educating patients and providers regarding risks of microdebrider usage. Elucidating the types of adverse outcomes associated with microdebrider usage and quantifying the likelihood of patient injury can help providers be better informed when using microdebriders in practice. Risk of the most common adverse events could be reduced if providers are familiarized with most common complications and learn from cases of operator error.

There are several limitations to our study. First, adverse events captured in this study were all self‐reported, increasing the risk for bias in recall and reporting. Second, there is limited standardization in submission of adverse events to the MAUDE database; submissions possess varying amounts of detail regarding specific device, manufacturer, procedure, and context. For example, the majority (59.2%) of analyzed adverse reported events did not specify the type of surgery in which the microdebrider was used. Additionally, although more adverse events were reported from 2012 to 2014 compared to other years studied, we could not find a temporal relationship due to lack of specification and standardization of the MAUDE database. For example, submissions often do not specify version or manufacturer of the microdebrider; this is a limitation as different manufacturers and models of microdebrider may be associated with distinct complication rates. Further studies with standardized reporting protocols are indicated to allow for a more detailed understanding of the risks of the microdebrider in clinical practice.

## Conclusion

Microdebriders are commonly used in a variety of otolaryngological procedures, including FESS and laryngotracheal procedures. However, use of microdebriders does pose some risk to patients. The most commonly reported injuries to patients in this study were unintentional tissue damage, burns, and bleeding. Patients also experienced ophthalmic damage and CSF leaks. The most common device malfunctions were broken pieces (burs, blades, handpieces), motor overheatings, and navigation system malfunctions. These device malfunctions, particularly broken pieces, were often associated with operator misuse. Risk of the most common adverse events could be reduced if providers are familiarized with most common complications and learn from cases of operator error. Thus, improving provider training on proper usage of microdebriders can reduce the risk of device malfunction and patient injury. This, coupled with greater knowledge of the types of injury related to microdebrider use that are most common, could result in reduced risk of injury to patients. Further studies with a standardized reporting protocol are indicated.

## Author Contributions

**Hari Magge**, concept, design, acquisition, analysis, interpretation of data, drafting of the manuscript, critical revision of the manuscript for important intellectual content, accountability for all aspects of the work; **Esther Lee**, concept, design, acquisition, analysis, interpretation of data, drafting of the manuscript, critical revision of the manuscript for important intellectual content, accountability for all aspects of the work; **Timothy B. Shaver**, concept, design, acquisition, analysis, interpretation of data, drafting of the manuscript, critical revision of the manuscript for important intellectual content, accountability for all aspects of the work; **Punam G. Thakkar**, concept, design, Critical revision of the manuscript for important intellectual content, accountability for all aspects of the work; **Ameet Singh**, concept, design, critical revision of the manuscript for important intellectual content, accountability for all aspects of the work.

## Disclosures

### Competing interests

None.

### Funding

None.
